# Crystal structure of the N-terminal domain of human Timeless and its interaction with Tipin

**DOI:** 10.1093/nar/gkx139

**Published:** 2017-02-25

**Authors:** Sandro Holzer, Gianluca Degliesposti, Mairi L. Kilkenny, Sarah L. Maslen, Dijana Matak-Vinkovíc, Mark Skehel, Luca Pellegrini

**Affiliations:** 1 Department of Biochemistry, University of Cambridge, Cambridge CB2 1GA, UK; 2 MRC Laboratory of Molecular Biology, Cambridge CB2 0QH, UK; 3 Department of Chemistry, University of Cambridge, Cambridge CB2 1EW, UK

## Abstract

Human Timeless is involved in replication fork stabilization, S-phase checkpoint activation and establishment of sister chromatid cohesion. In the cell, Timeless forms a constitutive heterodimeric complex with Tipin. Here we present the 1.85 Å crystal structure of a large N-terminal segment of human Timeless, spanning amino acids 1–463, and we show that this region of human Timeless harbours a partial binding site for Tipin. Furthermore, we identify minimal regions of the two proteins that are required for the formation of a stable Timeless–Tipin complex and provide evidence that the Timeless–Tipin interaction is based on a composite binding interface comprising different domains of Timeless.

## INTRODUCTION

DNA replication in eukaryotes is performed by a large and dynamic protein assembly known as the replisome (1). The replisome contains the necessary enzymatic activities required to unwind the double-stranded DNA, prime and perform bulk DNA synthesis and process Okazaki fragments on the lagging-strand template. The normal progress of DNA replication can be challenged by alterations in the DNA template, such as DNA damage and unusual secondary structures, or the presence of protein–DNA barriers, which can lead to replication fork stalling and possible collapse, eventually threatening genomic integrity (2). To cope with such eventuality, the replisome is equipped with non-enzymatic components that are not directly involved in DNA synthesis, but are thought to be essential for maintaining replisome integrity and modulating replisome function during replicative stress (3).

One such replisome component is Timeless, which was originally identified as a mammalian homologue of the *Drosophila* circadian clock protein TIM (4). Insight into its function in DNA replication came from the observation that its yeast orthologue Tof1 is a core component of the replisome, required for the DNA damage checkpoint during S-phase (5), for fork stalling during perturbed DNA replication and fork pausing at replication barriers (6,7). Timeless displays an evolutionary-conserved interaction with another replisome component, Tipin (8,9); Tipin and its yeast orthologue Csm3 have been ascribed roles in DNA synthesis that mirror those of Tof1 (7,10–12). Following these initial observation, experimental evidence has established that the metazoan Timeless–Tipin complex has important roles in genotoxic stress resistance and checkpoint activation (13–15).

During DNA replication, the Timeless–Tipin complex is part of a larger functional assembly known as the replication pausing (or protection) complex (RPC) that includes Claspin and AND-1 (Mrc1 and Ctf4 in budding yeast, respectively) (3). Beyond its function in replication, Timeless has an important role in sister chromatid cohesion (16). Recently reported interactions of Timeless with Parp1 and Dbf4-dependent kinase suggest further roles in recombinational repair and coordination of homologous recombination with meiotic DNA replication, respectively (17,18).

Despite the central role of Timeless in DNA replication and maintenance of genomic stability, there is a lack of structural and biochemical data for the complex, needed to improve our mechanistic understanding of its function. Current structural information is limited to a low-resolution electron microscopy map of Timeless–Tipin in complex with single-stranded DNA-binding (19) and a crystal structure of a small C-terminal domain of Timeless bound to DNA repair protein PARP1 (17). Here we present a high-resolution structure of a large N-terminal region of human Timeless and provide a biochemical and biophysical characterization of the human Timeless–Tipin complex.

## MATERIALS AND METHODS

### Protein expression and purification

The sequences for the human Timeless constructs were amplified from I.M.A.G.E cDNA clone IRATp970C0576D (Source Bioscience). Using BamHI and NotI restriction sites, Timeless constructs were cloned into MCS I of a pRSF-Duet derived vector that contains a 14xHis-Sumo tag upstream of MCS I. The human Tipin Open Reading Frame (ORF) was codon-optimized for *Escherichia coli* expression and synthesized by Life Technologies. NcoI and XhoI restriction sites were used to clone the Tipin constructs into a pGAT3 vector (20) resulting in a N-terminal TEV-cleavable 6xHis-GST-tag for Tipin.

Either Timeless constructs alone or Timeless and Tipin constructs were co-transformed into Rosetta2(DE3) (Novagen) competent cells for protein expression. Bacterial cultures were grown in 2xYT media at 37°C to OD_600_: 0.6, then the temperature was reduced to 20°C and cultures were induced with 0.3 mM isopropyl β-D-1-thiogalactopyranoside (IPTG). The cells were harvested after 16 h incubation at 20°C. Ethylenediaminetetraacetic acid (EDTA)-free protease inhibitor tablet (Sigma Aldrich) and 25 units benzonase (Merck Millipore) in 15 ml of 25 mM Hepes pH 7.2, 150 mM KCl were used to resuspend each cell pellet yielding from 1 l bacterial culture, before lysis by sonication. The lysate was cleared by centrifugation at 10°C and 40 000 *g* for 1 h. After centrifugation the lysate was filtered with a 5 μm syringe filter. Subsequently proteins were purified in three consecutive steps: Ni-NTA agarose (Qiagen), HiTrap Q HP (GE Healthcare) and HiLoad 16/600 Superdex 200 pg (GE Healthcare). For nickel affinity purification the cleared and filtered lysate was subjected to 3 ml of Ni-NTA agarose in a gravity-flow column. The resin was washed by 6 column volumes of wash buffer (Hepes pH 7.2, 150 mM KCl), followed by 12 column volumes of wash buffer supplemented with 40 mM imidazole. A total of 10 ml of Hepes pH 7.2, 150 mM KCl, 200 mM Imidazole was used to elute the bound protein. The elution was applied onto a 5 ml HiTrap Q HP column and subsequently washed with 4 column volumes of wash buffer. For elution, a salt gradient from 150 to 500 mM KCl over the course of 10 column volumes was performed. Using a 10 kDa MWCO spin concentrator, the elution sample was concentrated to a volume of 1 ml and loaded onto a HiLoad 16/600 Superdex 200 pg size exclusion chromatography (SEC) column equilibrated in 25 mM Hepes 7.2, 150 mM KCl.

Full-length Timeless fused to an N-terminal twin-strep tag (IBA Life Sciences) and Tipin fused to an N-terminal 10xHis tag were cloned into a pFBDM (Geneva Biotech) vector using BamHI/NotI and XhoI/KpnI restriction sites, respectively. Subsequently, the plasmid was transformed into DH10MultiBac cells for bacmid generation, and the baculovirus for full-length Timeless and Tipin expression was generated as outlined in the MultiBac protocol (Geneva Biotech). Sf9 insect cells were transfected with the recombinant baculovirus at a multiplicity of infection (MOI) of 1 and harvested after 3 days. Cell pellets from 1 l Sf9 culture were resuspended in 15 ml of 25 mM Hepes pH 7.2, 150 mM KCl, 5% glycerol, 0.1% Triton X100 supplemented with EDTA-free protease inhibitor tablet (Sigma Aldrich) and 25 units benzonase (Merck Millipore). Lysis, centrifugation and nickel-affinity chromatography was performed as described above for Timeless and Tipin constructs expressed in bacteria, but using a buffer supplemented with 5% glycerol. The eluted sample from the nickel affinity purification was incubated for 10 min with 2 ml of Strep-Tactin resin (IBA Life Sciences), washed in a gravity flow column with 15 column volumes of buffer and eluted in 4 × 2 ml of buffer and 5 mM desthiobiotin. The purest fractions were pooled and concentrated to 1 ml and subjected to SEC using HiLoad 16/600 Superdex 200 pg column (GE Healthcare) in 25 mM Hepes pH 7.2, 150 mM KCl, 5% glycerol.

### Crystallization and crystal structure determination

Timeless 1–463 (Tim_N_) was crystallized by vapour diffusion at 19°C, mixing 200 nl protein with 200 nl of reservoir solution, consisting of 20% (w/v) polyethylene glycol (PEG) 3350, 200 mM Na_2_SO_4_. For crystallization the protein was concentrated to 8.5 mg/ml in the SEC buffer and 1 mM tris(2-carboxyethyl)phosphine (TCEP) was added. To cryoprotect the crystals the mother liquor was replaced stepwise by a 2:1:1 (volume ratio) mixture of reservoir solution, SEC buffer and 100% glycerol. Heavy-metal derivative crystals were obtained by 30 min incubation in the cryoprotectant mix in the presence of 5 mM K_2_PtCl_4_, and a heavy-metal derivative dataset was collected at I02 beamline of the Diamond Synchrotron. The X-ray diffraction data were indexed, scaled and integrated with the XDS software package (21) and the structure was solved using the anomalous signal of the K_2_PtCl_4_-soaked crystals using PHASER (22). Inspection of the 2.23 Å electron density map for a near-complete model of Tim_N_ showed that density for amino acids 239–330 was missing, likely due to proteolysis during crystallization. A new Tim_N_ construct where residues 239–330 were replaced with a 6-residue linker sequence GSTGST was prepared, which crystallized more readily and yielded larger crystals. X-ray diffraction data for crystals of TimN_Δ239-330_ was collected at Proxima 1 beamline of the Soleil Synchrotron and solved by molecular replacement in PHASER using the Platinum derivative structure as a search model. The model was subsequently completed by iterative cycles of manual building in Coot (23) and crystallographic refinement in Phenix (24).

### Size-exclusion chromatography multi-angle laser light scattering (SEC-MALLS)

For SEC-MALLS experiments a Superdex 200 Increase 10/300 column (GE Healthcare) was coupled with a DAWN HELEOS II MALLS detector with a 664 nm laser light source, eight fixed-angle detectors and an Optilab T-rEX differential refractometer (Wyatt Technology) at 25°C. A total of 1–2 mg/ml (100 μl injection) of Timeless(1–1208_Δ239-330_) and Timeless(1–463_Δ239-330_) were analysed in 25 mM Hepes 7.2, 150 mM KCl, 50 mM Arginine, 50 mM Glutamate. The collected data were analysed and processed using ASTRA 6 (Wyatt Technology).

### Size-exclusion chromatography small-angle X-ray scattering (SEC-SAXS)

A Superdex 200 Increase 3.2/300 column (GE Healthcare) was attached to the HPLC system at the SWING beamline at Soleil Synchrotron. A total of 50 μl protein sample at 5 mg/ml was injected onto the column. To achieve satisfactory sample homogeneity, the experiment was performed in the following buffer: 25 mM Hepes 7.2, 150 mM KCl, 50 mM Arginine, 50 mM Glutamate. The scattering data was processed with the FOXTROT software as implemented at the SWING beamline, and the data analysis was performed using ScÅtter 3.0i (http://www.bioisis.net/).

### Pull-down assays

Timeless and Tipin constructs were transformed into Rosetta2(DE3) (Novagen) and 10 ml cultures grown to OD_600_ of 1. After induction with 0.3 mM IPTG, the cultures were incubated at 20°C for 16 h with constant shaking at 220 rpm. Cells were lysed by sonication in 25 mM Hepes 7.2, 150 mM KCl and the lysate was treated with protease inhibitors and benzonase. Subsequently the cleared lysate was applied to 50 μl of Glutathione Sepharose 4B (GE Healthcare). After three consecutive 1 ml washes with buffer, the proteins were eluted with 20 mM reduced glutathione and analysed by sodium dodecyl sulphate-polyacrylamide gel electrophoresis.

### Mass spectrometry and cross-linking

Purified Timeless–Tipin complex (2 mg/ml) was cross-linked with isotopically-coded *N*-hydroxysuccinimide ester disuccidinimidyl suberate (DSS H_12_/D_12_) (Creative Molecules, Canada) at a final concentration of 0.22 mg/ml. The reaction was incubated for 45 min at 37 °C and quenched by adding NH_4_HCO_3_ to a final concentration of 50 mM and incubating for further 15 min.

The cross-linked sample was freeze-dried and resuspended in 50 mM NH_4_HCO_3_ to a final protein concentration of 1 mg/ml, reduced with 10 mM dithiothreitol (DTT) and alkylated with 50 mM iodoacetamide. Following alkylation, proteins were digested with trypsin (Promega, UK) at an enzyme-to-substrate ratio of 1:20, overnight at 37°C. The sample was acidified with formic acid to a final concentration of 2% (v/v) and the peptides fractionated by peptide SEC, using a Superdex Peptide 3.2/300 column (GE Healthcare) with 30% (v/v) acetonitrile/0.1% (v/v) trifluoroacetic acid (TFA) as mobile phase and at a flow rate of 50 μl/min. Fractions were collected every 2 min over the elution volume 1.0 to 1.7 ml, lyophilized and resuspended in 2% (v/v) acetonitrile and 2% (v/v) formic acid.

The fractions were analysed by nano-scale capillary LC-MS/MS using an Ultimate U3000 HPLC (ThermoScientific Dionex, USA) to deliver a flow of approximately 300 nl/min. A C18 Acclaim PepMap100 5 μm, 100 μm × 20 mm nanoViper (ThermoScientific Dionex, USA), trapped the peptides before separation on a C18 Acclaim PepMap100 3 μm, 75 μm × 250 mm nanoViper (ThermoScientific Dionex, USA). Peptides were eluted with a gradient of acetonitrile. The analytical column outlet was directly interfaced via a nano-flow electrospray ionization source, with a hybrid dual pressure linear ion trap mass spectrometer (Orbitrap Velos, ThermoScientific, USA). Data-dependent analysis was carried out, using a resolution of 30 000 for the full mass spectrometry (MS) spectrum, followed by ten MS/MS spectra in the linear ion trap. MS spectra were collected over a *m/z* range of 300–2000. MS/MS scans were collected using threshold energy of 35 for collision-induced dissociation.

For data analysis, Xcalibur raw files were converted into the open mzXML format through MSConvert (Proteowizard) with a 32-bit precision. mzXML files were directly used as input for xQuest searches on a local xQuest installation (25). The selection of cross-linked precursor MS/MS data were based on the following criteria: a mass shift of 12.07532 Da among the heavy and the light cross-linker; precursor charge ranging from 3+ to 8+; maximum retention time difference 2.5 min. Searches were performed against an *ad hoc* database containing the Timeless and Tipin sequences. The following parameters were set for xQuest searches: maximum number of missed cleavages (excluding the cross-linking site) 3; peptide length 4–50 amino acids; fixed modifications carbamidomethyl-Cys (mass shift 57.02146 Da); mass shift of the light cross-linker 138.06808 Da; mass shift of mono-links 156.0786 and 155.0964 Da; MS1 tolerance 10 ppm, MS2 tolerance 0.2 Da for common ions and 0.3 for cross-link ions; search in enumeration mode (exhaustive search). Search results were filtered according to the following criteria: MS1 mass tolerance window −3 to 7 ppm. Finally each MS/MS spectra was manually inspected and validated.

### Non-dissociative mass spectrometry

Non-denaturing nanoESI–MS Spectra of the Timeless–Tipin complex were recorded on a Synapt HD mass spectrometer (Waters) modified for studying high masses. Protein samples were exchanged into 0.20 M ammonium acetate (pH 7.0) solution using MicroBioSpin 6 chromatography columns (BioRad) and diluted to a final concentration of 5–10 μM before analysis. A total of 2.5 μl of protein solution was electrosprayed from a borosilicate emitter (Thermo Scientific) for sampling. Typical conditions were capillary voltage 1.6–2.2 kV, cone voltage 60–112 V, Trap 12–28 V, Transfer 12 V with backing pressure 3–4 mbar and source temperature of 20°C. Spectra were calibrated externally using cesium iodide. Data acquisition and processing were performed using MassLynx 4.1.

## RESULTS

### Crystal structure of an N-terminal region of Timeless

Human Timeless is a large, multi-domain protein of 1208 amino acids. In a systematic effort to identify regions of the protein that could be amenable to crystallization, we produced a construct of Timeless spanning residues 1–463, that was highly overexpressed and soluble in recombinant form. We established crystallization conditions for this construct, hereafter referred to as Tim_N_, that yielded crystals suitable for X-ray diffraction analysis. As no homologous structure was available in the protein data bank (PDB) that could be used as template for molecular replacement, the structure was determined using the anomalous signal in diffraction data of crystals soaked with K_2_PtCl_4_. Inspection of the 2.2 Å structure revealed that a large segment of the protein construct, spanning residues 239–330, was not visible in the electron density map, presumably because of conformational disorder. Consequently, a new Tim_N_ construct was prepared carrying an internal deletion of residues 239–330, that crystallized more readily and yielded crystals diffracting to a higher resolution of 1.85 Å (Supplementary Table S1). Removal of residues 239–330 did not cause structural alterations in either the Tim_N_ crystal structure, or the solution shape of the Timeless–Tipin complex; small-angle X-ray scattering analysis showed a small but significant decrease in hydrodynamic radius for the complex missing Timeless residues 239–330, indicating that these amino acids are disordered in solution (Supplementary Figures S1 and 2).

**Figure 1.**
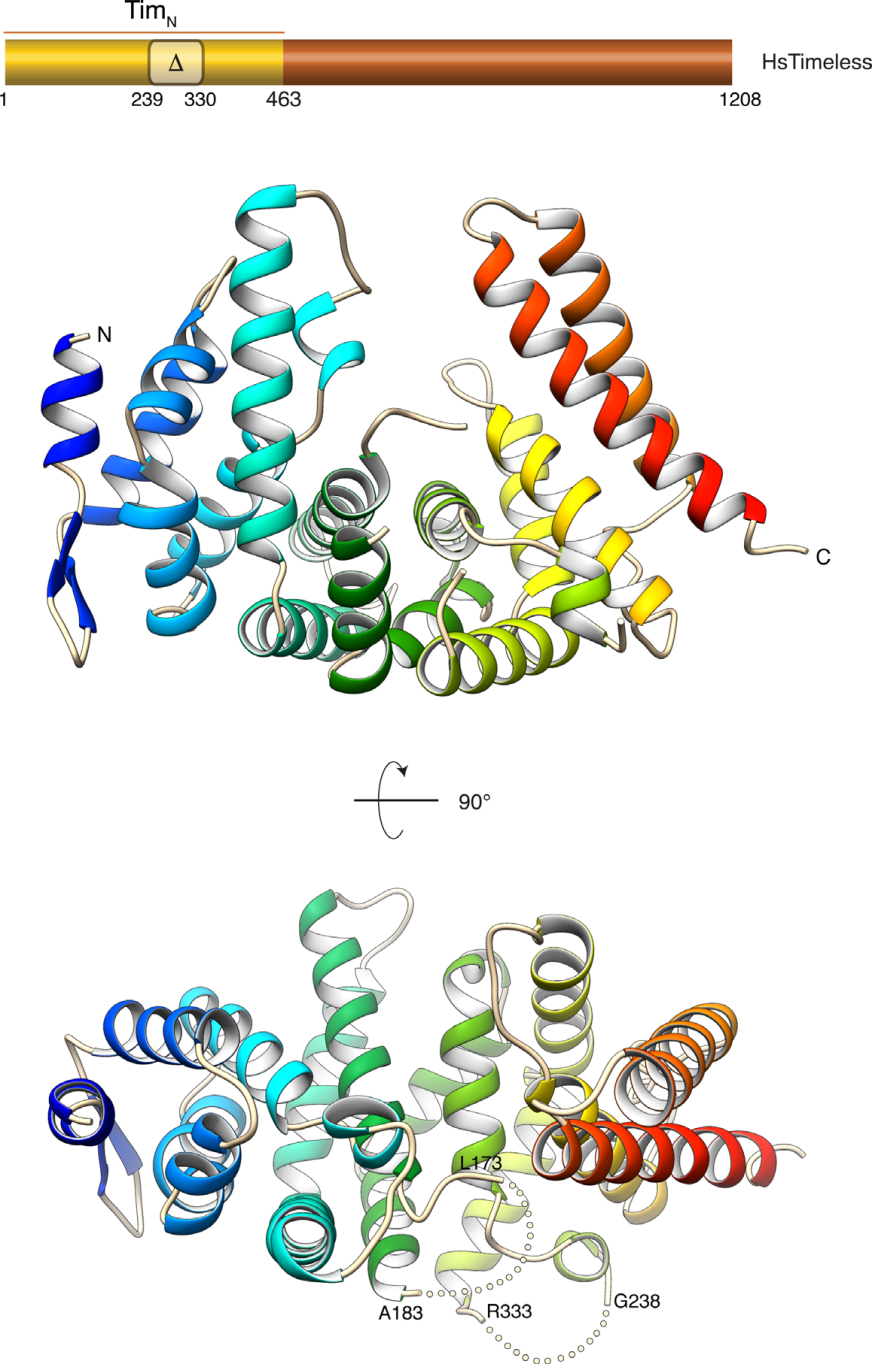
Crystal structure of amino acids 1–463 of human Timeless (Tim_N_). The structure is drawn as a ribbon, coloured blue (N-terminus) to red (C-terminus). Two views of the structure are shown. An indication of the region of human Timeless solved by X-ray crystallography is provided in the diagram of the full-length protein, above the ribbon drawings. The region of amino acids 239–330 that was removed in the Tim_N_ construct which yielded better crystals is indicated. The position of the two disordered loops in the Tim_N_ structure is highlighted by dotted lines in the bottom view of the structure.

The N-terminal domain of Timeless is an α-helical protein with an overall shape that is reminiscent of a horseshoe (Figure 1). Although it is not possible to assign Tim_N_ to one of the well-known classes of helical-repeat proteins such as the HEAT or ARM repeat families, Tim_N_ does contain six two-helix hairpins, with the N-terminal four hairpins containing an additional helix in the intervening segment between the first and the second helix of the hairpin. Surprisingly, Tim_N_ forms a dimer in solution and in the crystal; however, SEC multi-angle laser scattering (SEC-MALLS) experiments showed that full-length Timeless exists as a monomer in solution (Supplementary Figure S3). It is likely therefore that the observed dimerization of recombinant Tim_N_ is an artifact of construct design.

**Figure 2.**
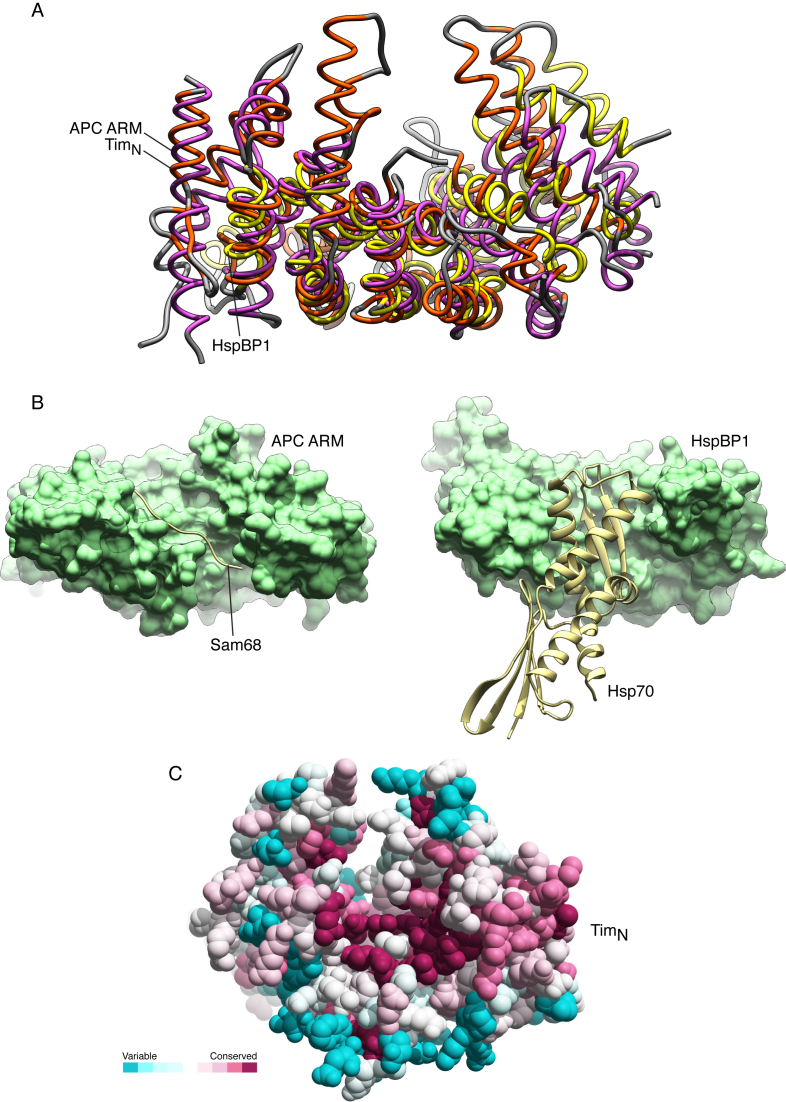
Structural similarity of Tim_N_ with protein domains that mediate protein–protein interactions. (**A**) Superposition of the Tim_N_ structure with the ARM domain of the Adenomatous Polyposis Coli (APC) protein (PDB ID: 3QHE) and the HspBP1 core domain (PDB ID: 1XQS). The protein structures are shown as thin tubes, and their secondary structure elements coloured orange (Tim_N_), purple (APC) and yellow (HspBP1). The fold similarity search was performed in PDBeFold (http://www.ebi.ac.uk/msd-srv/ssm/) and the superposition in UCSF Chimera (31). (**B**) Side-by-side view of the structures of APC ARM domain bound to Sam68 (PDB ID: 3QHE) (left) and HspBP1 bound to the adenosine triphosphatase domain of Hsp70 (PDB ID: 1XQS) (right). APC and HspBP1 are shown in solvent-accessible surface representation in light green, and their ligands as ribbons in pale yellow. (**C**) Spacefill model of the Tim_N_ structure, with amino acids coloured according to a conservation score calculated by the ConSurf server (http://consurf.tau.ac.il/2016/). The colour scheme highlights the relative amino acid conservation, from turquoise (variable) to maroon (conserved).

It is unclear at present whether Tim_N_ mediates any of the numerous interactions reported for Timeless, including Tipin binding. The peculiar shape of the Tim_N_ structure suggests that its concave surface might act as a site of protein–protein interactions. Indeed, a search of the PDB for similar folds highlighted examples of Tim_N_-like proteins acting as receptors for ligands that bound to their concave face (Figure 2A). Two different ligand-binding modes are observed: in the case of the ARM domain of the Adenomatous Polyposis Coli (APC) protein bound to the tyrosine-rich domain of Sam68 (PDB ID: 3QHE), the ligand binds in extended conformation across the concave groove of APC (26), whereas in the case of the HspBP1 core domain in complex with Hsp70 (PDB ID: 1XQS), the adenosine triphosphatase domain of Hps70 docks into the concave groove of HspBP1 (27) (Figure 2B). In agreement with these observations, evolutionary conservation of surface residues in the Tim_N_ structure point to a higher degree of conservation for residues lining the concave groove, relative to the rest of the Tim_N_ structure (Figure 2C).

### Biophysical and biochemical characterization of the Timeless–Tipin complex

**Figure 3.**
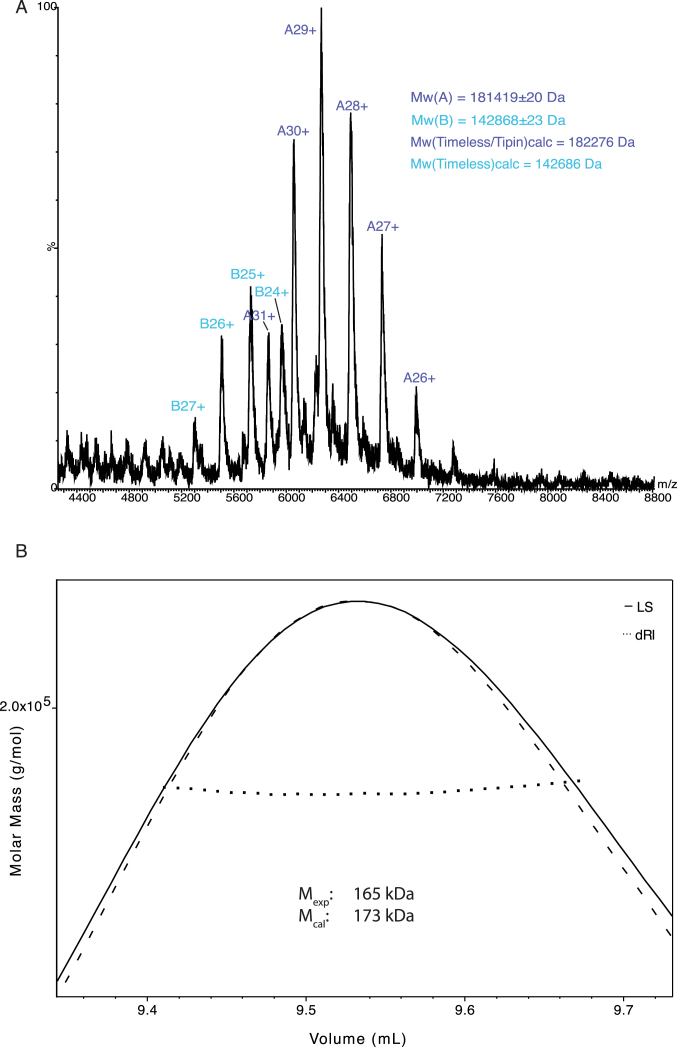
The human Timeless–Tipin complex exists in solution as a 1:1 heterodimer. (**A**) Non-dissociative mass spectrometry of the Timeless–Tipin complex. (**B**) Analysis by size-exclusion chromatography multi-angle laser scattering of the Timeless–Tipin complex.

The Timeless–Tipin complex was reconstituted by co-expression in either insect cells or bacteria and purified to homogeneity. Co-expression of Timeless and Tipin was necessary, as it proved to be impossible to purify Tipin on its own, due to poor biochemical behaviour likely caused by lack of its constitutive partner. Only limited amounts of full-length Timeless could be prepared, as the sample was difficult to handle biochemically. A 1:1 stoichiometry of the Timeless–Tipin complex was determined by non-dissociative MS and MALS, in agreement with previous observations (19) (Figure 3).

**Figure 4.**
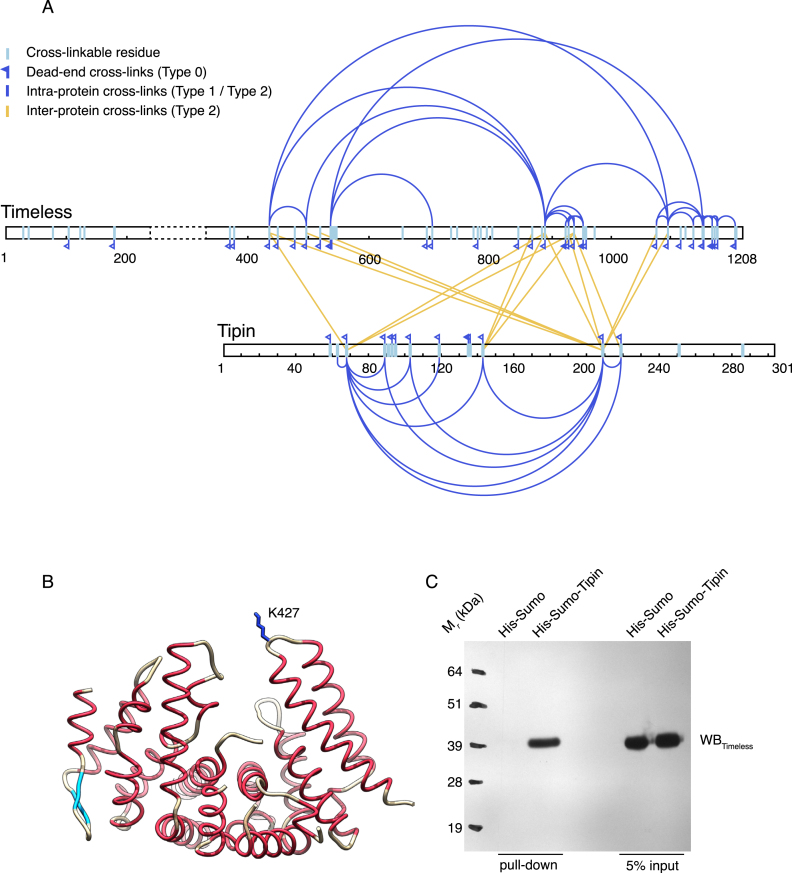
Cross-linking mass spectrometry (XL-MS) analysis of the human Timeless–Tipin complex. (**A**) Result of the XL-MS analysis (see also Supplementary Table S2). (**B**) Drawing of the Tim_N_ structure, highlighting the position of residue K427 that crosslinks with Tipin in the XL-MS analysis. (**C**) Western blot of a pull-down experiment showing that Tim_N_ is retained by His-Sumo-Tipin bound to Ni-NTA beads.

In order to begin to elucidate the structural basis for the interaction between Timeless and Tipin, we performed a cross-linking MS (XL-MS) analysis of the Timeless–Tipin complex. Based on a lysine-specific cross-linker, the XL-MS experiment showed that the Timeless–Tipin interaction is clearly complex and extensive: three distinct regions of Timeless between amino acids 400 and 1100 cross-linked to three different sites in Tipin (Figure 4A and Supplementary Table S2). Conversely, the region of Tipin involved in inter-molecular crosslinks with Timeless consisted largely of its central, helical core and did not involve the flanking regions, that are predicted to be mostly disordered.

Several long-range intra-protein cross-links were observed in the interacting regions of both Timeless and Tipin. These cross-links involved the same lysines observed in inter-protein cross-links, indicating that distant regions in the primary structure of the two proteins are in close proximity in the tertiary structure defining the binding interface. Furthermore, the observation that Tipin can contact regions of Timeless that are separated by hundreds of amino acids suggests that, in the complex, Timeless forms a binding surface capable of surrounding the smaller protein Tipin.

Timeless residue K427, retained in the Tim_N_ construct and in a suitable position to contact a ligand occupying Tim_N_'s concave groove, is involved in two inter-molecular cross-links with lysines 66 and 207 of Tipin (Figure 4B and Supplementary Table S2). Pull-down experiments showed that Tim_N_ can support direct binding to Tipin, although the interaction does not lead to a stable association as observed for full-length Timeless (Figure 4C). In order to determine the core region of both proteins required to support wild-type association, we performed a systematic search for the minimal constructs in each protein partner that would still retain wild-type association. A set of different constructs for Timeless and Tipin was therefore generated, to determine which parts of the proteins are required for complex formation. For this experiment, we used a version of Timeless missing amino acids 239–330 that were found to be disordered in the Tim_N_ crystal structure, as removal of this region improved greatly the biochemical behaviour of the recombinant protein.

**Figure 5.**
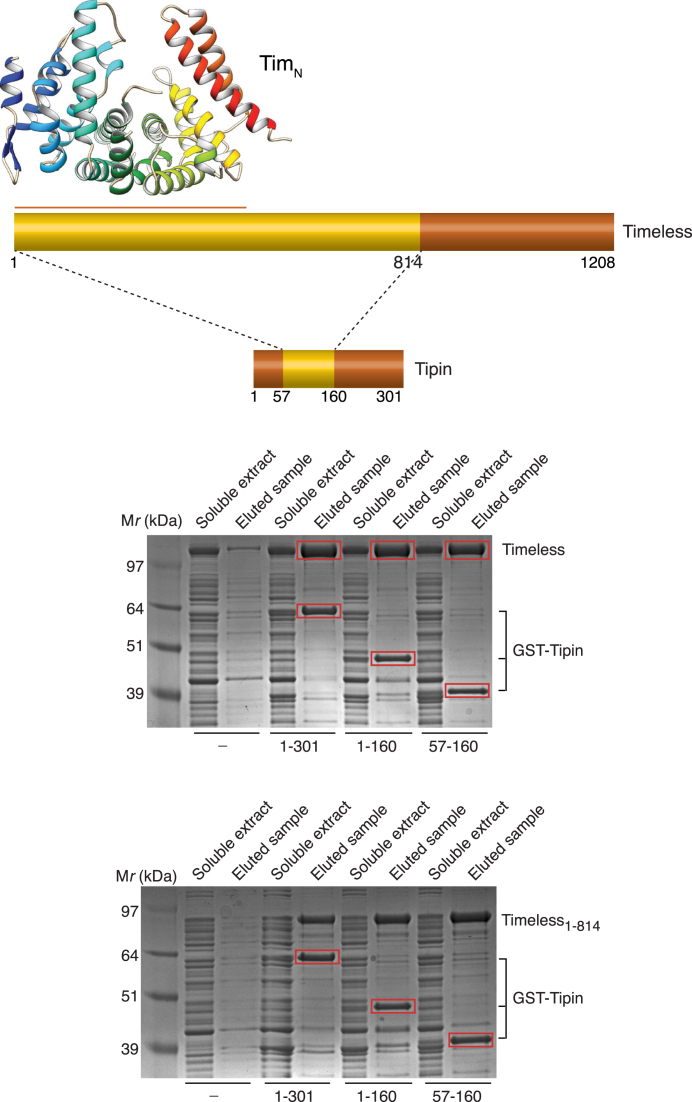
Determination of the minimal interacting regions of Timeless and Tipin. Various constructs for Timeless (1–1208, 1–814) and Tipin (1–301, 1–160 and 57–160) were co-expressed in bacteria and their interaction was tested by recovery and elution from glutathione sepharose beads of GST-tagged Tipin.

Based on pull-down assays, we showed that it is possible to reconstitute a stable interaction between amino acids 1–814 of Timeless and amino acids 57–160 of Tipin (Figure 5). Thus, the binding site for Tipin is contained within a large N-terminal region of Timeless, which is predicted to be highly structured and mostly helical in content; conversely, the C-terminal residues of Timeless, which contains the recently identified Parp-binding domain (17,28), are not required for the interaction with Tipin. The Timeless-binding region of Tipin is contained within its central core, also predicted to be helical, whereas its N- and C-terminal tails are dispensable for the interaction.

## DISCUSSION

The findings reported here provide a first, partial high-resolution description of Timeless and a biochemical analysis of its interaction with Tipin, which will be necessary to understand the important role of this complex in maintaining genomic stability during DNA replication.

The biochemical basis for the function of the Timeless–Tipin complex in DNA replication remains obscure, despite the complex being an integral component of the eukaryotic replisome. Recently, a role for the yeast orthologue Tof1–Csm3 complex was proposed in avoiding DNA damage caused by excessive fork rotation during DNA replication (29), and increasing *in vitro* rates of DNA synthesis by a reconstituted yeast replisome (30). As the complex appears void of enzymatic activities, it is likely that its role involves mediating multiple interaction with constitutive and transient components of the replisome, as well as possibly with DNA. Although the Timeless–Tipin complex is considered part of a larger complex, known as the RPC, we have been unable to detect binary interactions of the Timeless–Tipin complex with RPC components Claspin and AND-1 using purified recombinant proteins (our unpublished data).

Our structural analysis shows that the N-terminal region of Timeless adopts a compact, highly helical fold that has been found to mediate protein–protein interactions in different physiological contexts (26,27); in agreement with these findings, we show that Tim_N_ can bind to Tipin in pull-down assays. The functional importance of this region of the protein is underscored by the presence of 37 missense mutations identified as putative cancer-promoting alterations in the Cosmic Database (http://cancer.sanger.ac.uk/cosmic) (Supplementary Figure S4). We further show that the minimal region of Timeless necessary for wild-type interaction with Tipin spans a larger N-terminal region that includes amino acids 1–814. We were not able to assess the contribution to Tipin binding of Timeless residues between 463 and 814 because of the poor solubility of these Timeless constructs.

Although the C-terminal residues of Timeless appear dispensable for a full-strength interaction with Tipin, mass-spectrometry coupled to cross-linking analysis of the complex shows that the C-terminal region of Timeless does come into close proximity with Tipin. Altogether, the evidence presented here supports a model of the Timeless–Tipin complex where the larger Timeless subunit binds its smaller Tipin partner via an extensive, composite interface to which distant regions of its primary sequence contribute, in agreement with previous low-resolution studies of the complex (19). How this complex architecture relates to Timeless–Tipin function will need to be elucidated by future structure–function experiments.

## ACCESSION NUMBER

The coordinates and structure factors for Tim_N_ have been deposited in the PDB under accession code 5MQI.

## Supplementary Material

Supplementary Data
