# Crystal structure of the N-terminal domain of human Timeless and its interaction with Tipin

**DOI:** 10.1093/nar/gkz1116

**Published:** 2019-11-22

**Authors:** Sandro Holzer, Gianluca Degliesposti, Mairi L Kilkenny, Sarah L Maslen, Dijana Matak-Vinkovíc, Mark Skehel, Luca Pellegrini

**Affiliations:** 1 Department of Biochemistry, University of Cambridge, Cambridge CB2 1GA, UK; 2 MRC Laboratory of Molecular Biology, Cambridge CB2 0QH, UK; 3 Department of Chemistry, University of Cambridge, Cambridge CB2 1EW, UK

*Nucleic Acids Research*, 2017, 45(9): 5555–5563, https://doi.org/10.1093/nar/gkx139.

In the purification protocol, the step of tag cleavage was inadvertently omitted. The Authors wish to make the following corrections to their article.

## MATERIALS AND METHODS

### Protein expression and purification

Current:

… A total of 10 ml of Hepes pH 7.2, 150 mM KCl, 200 mM Imidazole was used to elute the bound protein. The elution was applied onto a 5 ml HiTrap Q HP column and subsequently washed with 4 column volumes of wash buffer…

New:

… A total of 10 ml of Hepes pH 7.2, 150 mM KCl, 200 mM Imidazole was used to elute the bound protein. The imidazole concentration in theeluate was diluted to 50 mM.Sumo-protease to a sample concentration of 0.02 mg/mL and 1mM DTT was added to the protein solution and incubated for 16 h at 4°C. The cleaved protein sample was applied onto a 5 ml HiTrap Q HP column and subsequently washed with 4 column volumes of wash buffer…

These corrections do not affect the results or conclusion of the article.

The published article has been updated.

