# Supplementary material for: Crystal structure of the N-terminal domain of human Timeless and its interaction with Tipin
Source: Nucleic Acids Res. 2017 Feb 25;45(9):5555–63. doi: 10.1093/nar/gkx139 (PMC5605233; doi:10.1093/nar/gkx139)
Supplement: Supplementary Data [file gkx139_Supp.pdf]

SUPPLEMENTARY DATA

Supplementary figure and table legend

Table S1 X-ray data collection and crystallographic refinement statistics.

Table S2 Protein cross-link summary of XL-MS analysis.

Figure S1 SAXS analysis of full-length human Timeless-Tipin complex. **A** Guinier plot. **B** Normalised Kratky plot. **C** P(r) distribution diagram.

Figure S2 SAXS analysis of the human Timeless-Tipin complex missing amino acids 239 to 330 of Timeless. **A** Guinier plot. **B** Normalised Kratky plot. **C** P(r) distribution diagram.

Figure S3 SEC-MALS analysis of Tim_N and Timeless, showing that Tim_N is a dimer in solution, but Timeless is monomeric. For these experiments, a version of Timeless lacking amino acids 239 to 330 was used, because of its improved biochemical behavior relative to the intact molecule.

Figure S4 Drawing of the Tim_N structure, showing the position of the cancer-associated missense mutations present in the Cosmic Database (http://cancer.sanger.ac.uk/cosmic).

Supplementary table 1. X-ray data collection and crystallographic refinement statistics

Data collection

Average unit cell: 68.01 71.34 185.05 90.00 90.00 90.00

Space group: $C\ 2\ 2\ 2_1$

	Overall	InnerShell	OuterShell
Low resolution limit	49.22	49.22	1.89
High resolution limit	1.85	9.05	1.85
Rmerge	0.033	0.015	0.752
Rmeas	0.037	0.018	0.867
Rpim	0.018	0.009	0.422
Total number of observations	140745	1232	7835
Total number unique	36572	338	2109
Mean((I)/sd(I))	18.6	59.4	1.6
Mn(I) half-set correlation CC(1/2)	0.999	0.999	0.645
Completeness	94.4	90.2	87.8
Multiplicity	3.8	3.6	3.7

Refinement

Reflections used in refinement	36567 (3524)
Reflections used for R-free	1916 (185)
R-work	0.1726 (0.3156)
R-free	0.1795 (0.3236)
Number of non-hydrogen atoms	3031
Macromolecules	2850
ligands	20
Protein residues	345
RMS(bonds)	0.015
RMS(angles)	1.29
Ramachandran favored (%)	97
Ramachandran allowed (%)	3.2
Ramachandran outliers (%)	0.29
Rotamer outliers (%)	0.32
Clashscore	2.29
Average B-factor	59.23
macromolecules	59.46
ligands	81.94
solvent	52.20

Statistics for the highest-resolution shell are shown in parentheses.

Protein 1	Protein 2	Pos1	Pos2	X-link Type	Score
Timeless	Tipin	882	207	inter-protein xl	42.68
Timeless	Tipin	427	207	inter-protein xl	42.25
Timeless	Tipin	1085	207	inter-protein xl	38.62
Timeless	Tipin	882	66	inter-protein xl	38.05
Timeless	Tipin	882	141	inter-protein xl	37.77
Tipin	Timeless	209	488	inter-protein xl	35.94
Timeless	Tipin	930	66	inter-protein xl	35.42
Tipin	Timeless	219	930	inter-protein xl	32.27
Timeless	Tipin	930	141	inter-protein xl	31.89
Tipin	Timeless	207	921	inter-protein xl	31.64
Timeless	Tipin	1066	207	inter-protein xl	31.37
Timeless	Tipin	511	207	inter-protein xl	31.21
Timeless	Tipin	861	141	inter-protein xl	26.04
Tipin	Timeless	66	427	inter-protein xl	24.26

Protein 1	Protein 2	Pos1	Pos2	X-link Type	Score
Tipin	Tipin	101	207	intra-protein xl	42.68
Tipin	Tipin	217	207	intra-protein xl	44.4
Tipin	Tipin	217	66	intra-protein xl	34.21
Tipin	Tipin	207	89	intra-protein xl	32.32
Tipin	Tipin	141	207	intra-protein xl	31.71
Tipin	Tipin	66	207	intra-protein xl	31.55
Tipin	Tipin	101	66	intra-protein xl	30.95
Tipin	Tipin	66	61	intra-protein xl	26.9
Tipin	Tipin	66	87	intra-protein xl	26.4
Tipin	Tipin	117	66	intra-protein xl	25.05
Tipin	Tipin	66	141	intra-protein xl	23.54
Timeless	Timeless	882	427	intra-protein xl	45.27
Timeless	Timeless	1085	427	intra-protein xl	44.31
Timeless	Timeless	882	921	intra-protein xl	42.51
Timeless	Timeless	882	488	intra-protein xl	41.32
Timeless	Timeless	930	921	intra-protein xl	40.86
Timeless	Timeless	1144	1142	intra-protein xl	38.96
Timeless	Timeless	882	1085	intra-protein xl	38.52
Timeless	Timeless	1066	1142	intra-protein xl	38.41
Timeless	Timeless	1158	1167	intra-protein xl	37.36
Timeless	Timeless	882	945	intra-protein xl	35.61
Timeless	Timeless	1158	1142	intra-protein xl	35.33
Timeless	Timeless	427	488	intra-protein xl	35.19
Timeless	Timeless	882	528	intra-protein xl	34.58
Timeless	Timeless	1085	1142	intra-protein xl	31
Timeless	Timeless	882	930	intra-protein xl	30.57
Timeless	Timeless	1127	1142	intra-protein xl	30.34
Timeless	Timeless	1127	1085	intra-protein xl	29.04
Timeless	Timeless	1158	1166	intra-protein xl	27.98
Timeless	Timeless	528	1142	intra-protein xl	27.26
Timeless	Timeless	1196	1166	intra-protein xl	26.6
Timeless	Timeless	921	945	intra-protein xl	25.8
Timeless	Timeless	1142	1166	intra-protein xl	25.67
Timeless	Timeless	1066	1085	intra-protein xl	24.65
Timeless	Timeless	696	528	intra-protein xl	23.84
Timeless	Timeless	916	945	intra-protein xl	23.82

Protein 1	Pos1	X-link Type	Score
Timeless	882	monolink	55.43
Timeless	1167	monolink	55.02
Timeless	177	monolink	51.21
Timeless	469	monolink	51
Timeless	511	monolink	50.64
Timeless	930	monolink	50.35
Timeless	921	monolink	47.94
Timeless	1158	monolink	47.93
Timeless	771	monolink	47.21
Timeless	528	monolink	47.17
Timeless	362	monolink	45.53
Timeless	1142	monolink	45.46
Timeless	1144	monolink	45.13
Timeless	696	monolink	44.92
Timeless	488	monolink	43.64
Timeless	441	monolink	43.31
Timeless	1198	monolink	42.75
Timeless	861	monolink	42.69
Timeless	1106	monolink	42.2
Timeless	427	monolink	41.86
Timeless	1066	monolink	41.7
Timeless	687	monolink	40.66
Timeless	945	monolink	40.14
Timeless	838	monolink	39.94
Timeless	1085	monolink	39.48
Timeless	369	monolink	38.62
Timeless	950	monolink	38.57
Timeless	530	monolink	38.49
Timeless	1162	monolink	37.96
Timeless	102	monolink	37.13
Timeless	1127	monolink	36.75
Tipin	207	monolink	56.41
Tipin	117	monolink	51.96
Tipin	66	monolink	47.4
Tipin	101	monolink	46.92
Tipin	93	monolink	44.73
Tipin	133	monolink	44.38
Tipin	217	monolink	43.18
Tipin	134	monolink	43.08
Tipin	141	monolink	42.24
Tipin	57	monolink	40.44
Tipin	91	monolink	38.06

Protein 1	Pos1	X-link Type	Score
Timeless	916	intralink	51.31
Timeless	1196	intralink	45.92
Timeless	1197	intralink	41.13
Timeless	1143	intralink	38.13
Tipin	87	intralink	45.58

Supplementary table 2

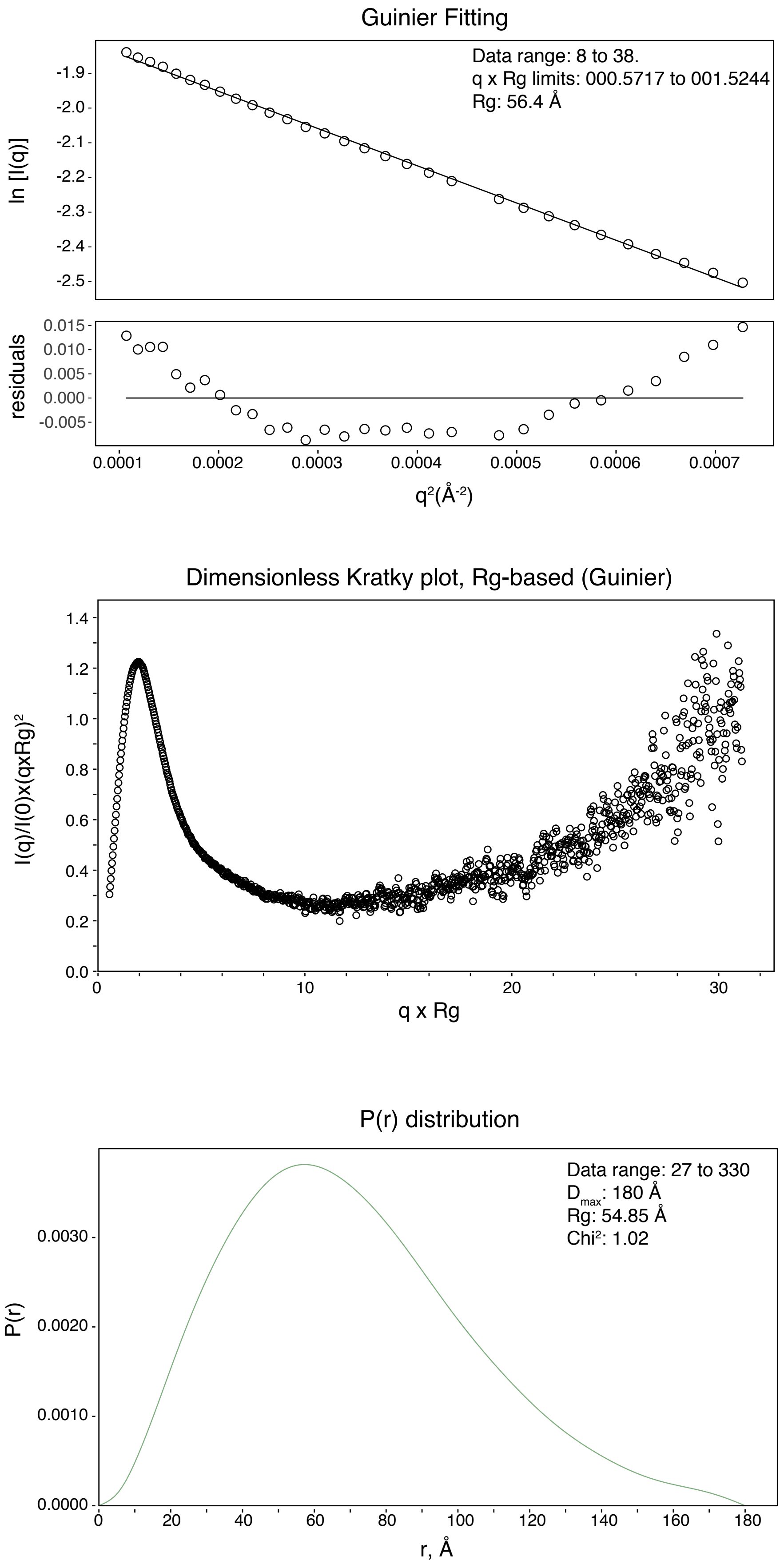

Supplementary figure 1

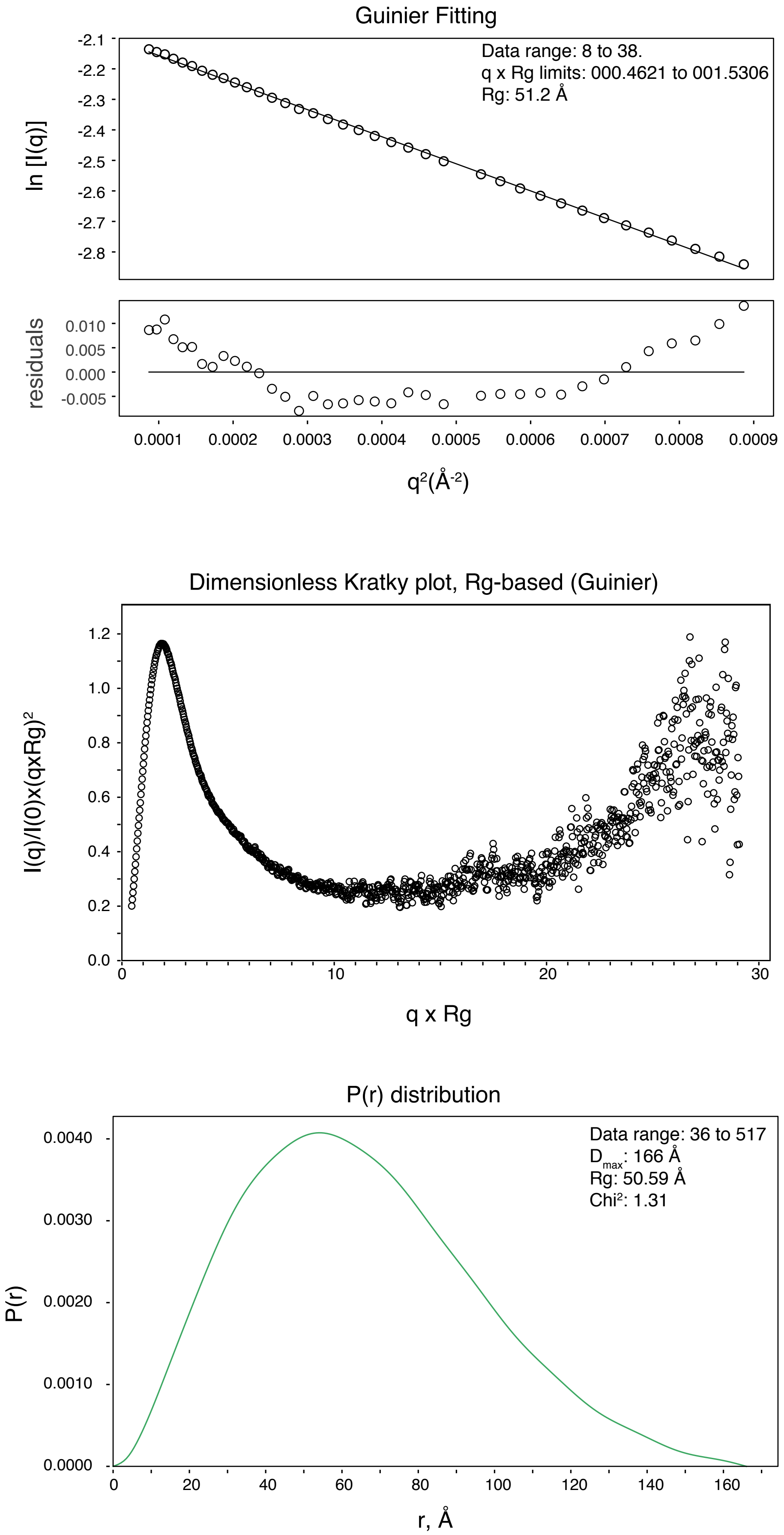

Supplementary figure 2

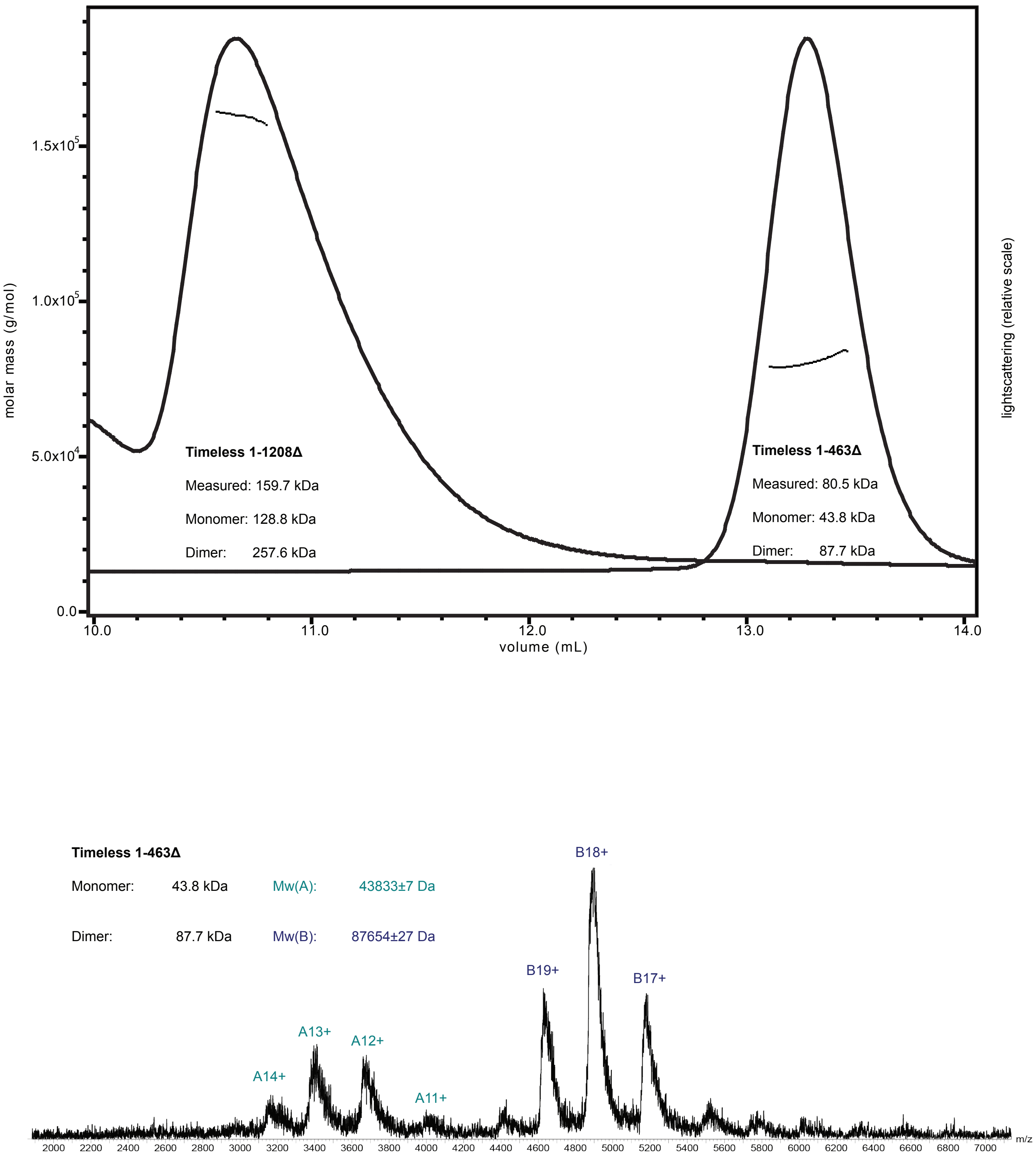

Supplementary Figure 3

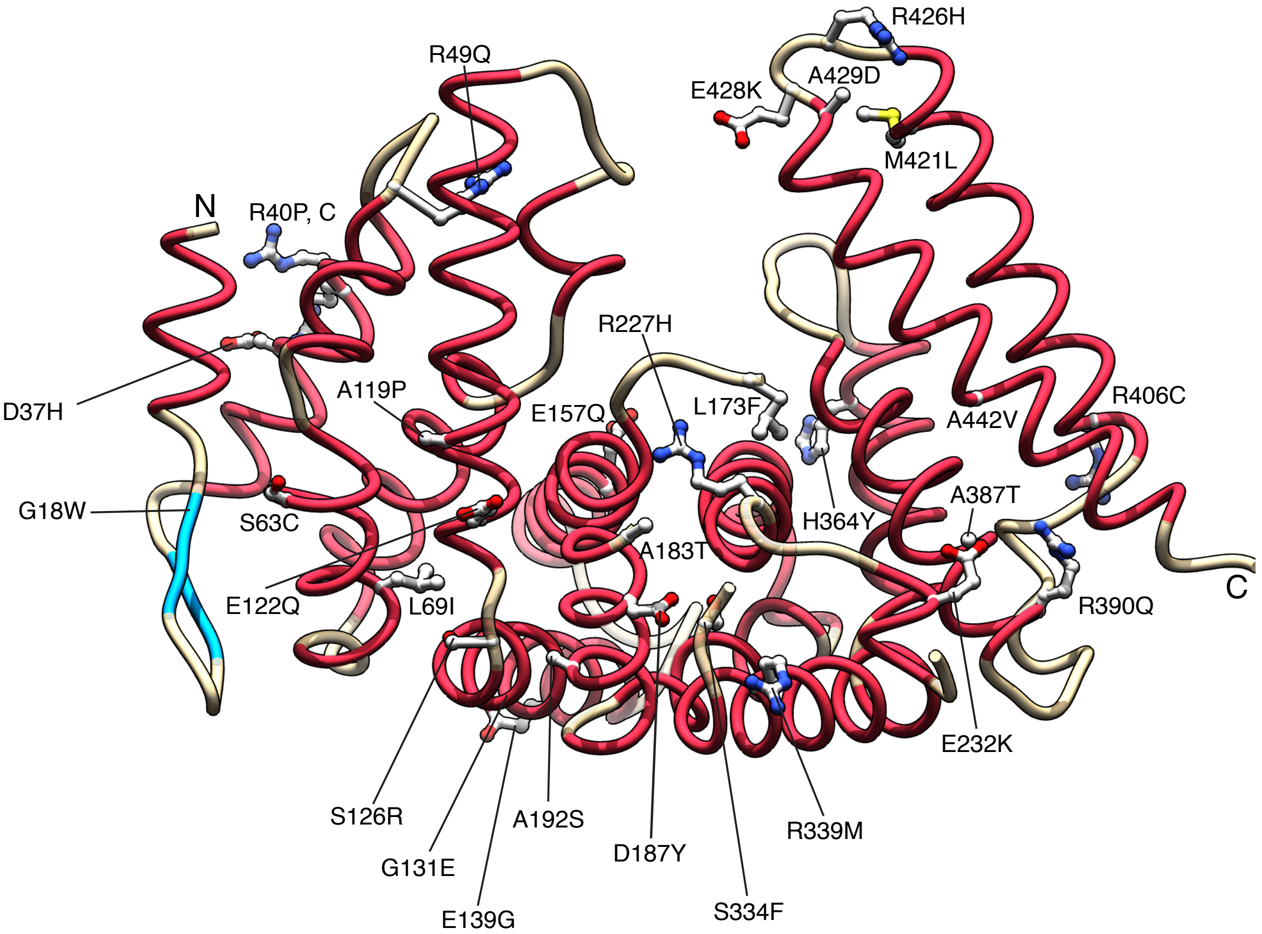

Supplementary Figure 4
